# Sleep and circadian rhythm disturbance in kidney stone disease: a narrative review

**DOI:** 10.3389/fendo.2023.1293685

**Published:** 2023-11-27

**Authors:** Si-Ke He, Jia-Hao Wang, Tao Li, Shan Yin, Jian-Wei Cui, Yun-Fei Xiao, Yin Tang, Jia Wang, Yun-Jin Bai

**Affiliations:** ^1^ Department of Urology, Institute of Urology, West China Hospital, Sichuan University, Chengdu, China; ^2^ Department of Urology, Affiliated Hospital of Guizhou Medical University, Guiyang, China; ^3^ Department of Urology, Affiliated Hospital of North Sichuan Medical College, Nanchong, China

**Keywords:** circadian rhythm disturbance, circadian clock, sleep disorder, circadian clock gene, kidney stone disease

## Abstract

The circadian rhythm generated by circadian clock genes functions as an internal timing system. Since the circadian rhythm controls abundant physiological processes, the circadian rhythm evolved in organisms is salient for adaptation to environmental change. A disturbed circadian rhythm is a trigger for numerous pathological events. Recently, accumulated data have indicated that kidney stone disease (KSD) is related to circadian rhythm disturbance. However, the mechanism between them has not been fully elucidated. In this narrative review, we summarized existing evidence to illustrate the possible association between circadian rhythm disturbance and KSD based on the epidemiological studies and risk factors that are linked to circadian rhythm disturbance and discuss some chronotherapies for KSD. In summary, KSD is associated with systemic disorders. Metabolic syndrome, inflammatory bowel disease, and microbiome dysbiosis are the major risk factors supported by sufficient data to cause KSD in patients with circadian rhythm disturbance, while others including hypertension, vitamin D deficiency, parathyroid gland dysfunction, and renal tubular damage/dysfunction need further investigation. Then, some chronotherapies for KSD were confirmed to be effective, but the molecular mechanism is still unclear.

## 1 Introduction

With the Earth’s planetary rotation, there is a 24-h oscillating light-dark cycle (1). To adapt to this environmental cycle, all animals and plants have evolved universal internal circadian rhythms (2). Such rhythms are observed in cellular, physiological, and biological behavioral processes within a 24-hour cycle. For instance, heart rate and body temperature increase in the morning and decrease in the evening (1, 3). It is also found in the diet, sleep-wake cycle, endocrine, absorption, and reproduction (4–7). Hence, circadian rhythm is of great significance for maintaining homeostasis and normal physiological activities.

Before artificial light was created, humans adjusted their lives to a natural day-night alteration cycle (8). With the great development of technology and society, life patterns have changed greatly, and the phenomenon of circadian rhythm disorder is now common due to social jet lag (SJL), shift work, and sleep disruption, which all contribute to abnormal daily rest/wake cycles and chronically disrupt endogenous circadian rhythms. The asynchrony between endogenous circadian rhythm and the sleep-wake cycle is defined as circadian rhythm disruption (9, 10). Growing epidemiological and genetic evidence shows that circadian disruption leads to various diseases, such as insomnia, hypertension, type 2 diabetes (T2D), chronic kidney disease (CKD), and even cancer (10–12). All of these factors finally cause immense loss of public health.

Kidney stone disease (KSD) is a common health concern with increasing incidence during the past few decades and occurs in a wide range of ages, including children, adolescents, and adults (13). The prevalence is approximately 10% globally, with a high recurrence rate of 50% within 5-10 years and 75% within 20 years (14–16). It causes such a large burden on public health since it is not only a transient acute symptom but is also linked to cardiovascular disease (CVD), CKD, end-stage renal disease (ESRD), and renal cancer (17, 18). An updated meta-analysis signifies that KSD is associated with an approximately 20%-40% higher risk for coronary artery disease, transient ischemia/stroke, and arterial disease (19). Then, the risks of ESRD, renal cell carcinoma, and transient cell carcinoma are all increased in patients with prior KSD history (18, 20). Although great progress has been achieved in traditional surgical management to provide better prognosis, the incidence and recurrence rates are still very high with little breakthrough in prevention methods (especially drug interventions) (21). Therefore, it is pivotal to explore the potential pathophysiological mechanisms of KSD to provide insights for prevention and therapy.

There is plenty of evidence to suggest that a circadian rhythm disturbance can promote KSD (22, 23). Herein, this review summarizes the relationship between circadian rhythm disturbance and KSD and discusses the potential mechanisms by which circadian rhythm affects KSD.

## 2 Biological characteristics of the circadian clock

The central circadian rhythm clock in the suprachiasmatic nucleus (SCN) is in the anterior part of the hypothalamus. It synchronizes with Earth’s time and feeds back to the downstream brain and peripheral regions by sympathetic nervous system transduction and hormone secretion after light signals from the light-dark cycle are received by the retina and transmitted to the SCN as electrical signals (10, 24). Some synchronization factors also called “time givers” or *zeitgebers* vary with temperature, diet, pharmacological manipulation, and social interactions (25). Additionally, peripheral organs, including the heart, liver, and kidney, participate in the “peripheral clock” and regulate cyclic physiological functions by manipulating the transcription of circadian genes, protein synthesis, energy metabolism, and so on. Both the central and peripheral clocks essentially share the same molecular structure, but the relationship between them remains unclear (10, 26).

**Figure 1:**
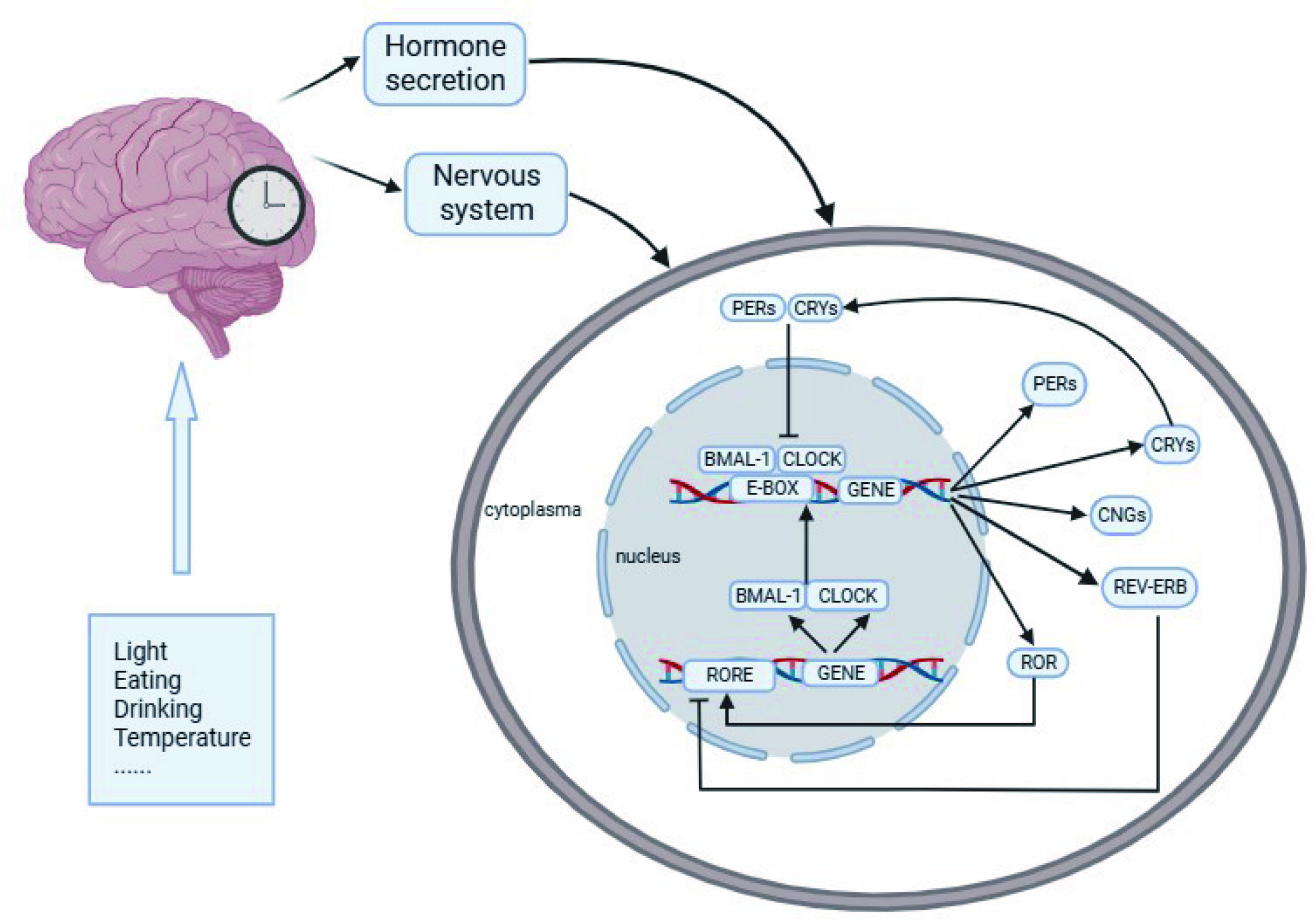
Molecular mechanism of the circadian rhythm. After cues from *Zeitgebers* of light, temperature, and feeding are perceived and transmitted to the SCN as electrical signals, the central circadian clock system will synchronize with geophysical time and feedback to the downstream brain regions and peripheral organs through the nervous system and hormone release. CLOCK and BMAL1 act as the center transcription factors of a heterodimer complex and activate the transcription of PER, CRY, REV-ERβ and RORα by cis-acting E-box and ROR elements. A multimeric complex formed by the PER/CRY proteins subsequently enters the nucleus to inhibit CLOCK/BMAL1 activity. Then, REV-ERBα and ROR proteins compete for response RORα/CLOCK/BMAL1 transcription. REV-ERBα reduces CLOCK/BMAL1 transcription, while RORα induces it. The main circadian genes are reactivated by the last with a new cycle beginning, and this feedback loop occurs at approximately 24 h.

At the molecular level, approximately 10% of genes are clock-controlled genes (CCGs) with circadian oscillations, also called circadian clock genes. The maintenance of circadian rhythm depends on a transcription-translation negative feedback loop formed by a series of interacting clock genes (25, 27, 28) (see **Figure 1**). The cycle of potential molecular mechanisms generating circadian rhythm is approximately 24 hours without synchronizing input; the central-peripheral network can adapt to a limited range of day lengths (29, 30). Some genes and regulators that are vital for initiating and maintaining circadian rhythm have been well investigated, such as the circadian locomotor output cycles kaput (*CLOCK*), a gene encoding the protein related to the length and persistence of a circadian circle (2); brain and muscle aryl hydrocarbon receptor nuclear translocator-like protein-1 coding gene (*BMAL1*), encoding the basic-helix–loop-helix transcription factor BMAL1 protein (2); the period family (*PER1/2/3*), key regulators in the cell cycle (31); cryptochrome 1&2 (*CRY1/2*), the main part of the negative feedback loop of the circadian clock and *REV-ERBα/β*, members of the orphan nuclear receptor family, which play a key role in regulating the expression of *CLOCK* and *BMAL1* (32, 33). These circadian clock genes regulate the day-night cycles by both positive and negative feedback cycles in the SCN and peripheral tissues and organs (10).

## 3 Mechanism of KSD

KSD is a common urological disease with a complicated pathological process. The major types include calcium stones (calcium oxalate [CaOx] & calcium phosphate [CaP]), uric acid (UA) stones (UAS), struvite stones (infection stones), and cystine stones. The biochemical process of stone formation is successive and complex and involves various physicochemical changes. Generally, four steps participate in pathogenesis: urinary supersaturation, crystal nucleation, growth, and aggregation. Crystal formation from supersaturated urine retained in the kidney is the driving force (16, 34). Then, the crystals gather together to grow to a size as further aggregation, which can interact with intrarenal structures (also called crystal-cell interaction) to cause renal tubular epithelial cell (RTEC) injury (34, 35). Crystal-cell interaction leads to the movement of crystals from the basolateral side of cells to the basement membrane and results in the retention of crystals in the kidney or collecting duct to eventually form the clinical stone (34, 36). Furthermore, a plaque of calcium deposited in the interstitial tissue of the renal papilla observed by electron microscopy, called Randall’s plaque (RP), appears to be the origin of urinary stones since it contributes greatly to crystal retention (37, 38). It should be noted that some factors are reported to be critical modulators for stone formation, including promoters and inhibitors. Promoters are receptors or receptor-like features that play vital roles in crystal−cell interactions for crystal retention in the kidney, while inhibitors can decrease crystallization and inhibit crystal aggregation and/or adhesion to RTECs. For example, serum calcium and vitamin D (vit D) act as promoters in KSD, while some metallic cations, such as magnesium, can inhibit crystal growth and aggregation (16, 39, 40). It is now widely accepted that stone formation usually depends on the imbalance between promoters and inhibitors.

## 4 Epidemiological evidence of circadian rhythm disruption in KSD

Light is the main *zeitgeber* that regulates the 24-h circadian cycle. Sleep and wakefulness can coregulate the circadian rhythm and maintain sleep homeostasis with light exposure. However, the day-night circadian rhythm has been altered greatly with the invention of incandescent lighting (2, 41). Circadian sleep disorder is defined medically as an inability to sleep at the desired time rather than an inability to sleep, for instance, staying up late working (night shift work) and rapidly travelling to new time zones (social jet lag [SJL]). Both shift work and SJL are popular and involve a large population and diverse professions (42, 43). Altered sleep-wake cycles disturb the internal circadian clock. Growing evidence shows that disordered circadian sleep and sleep insufficiency are closely related to multiple disorders, including KSD. Yin et al. (44) investigated the relationship between sleep status and KSD risk in a cross-sectional study and found that short sleep duration (< 7 h) was associated with a higher KSD prevalence than normal sleep duration (7-9 h) (odds ratio [OR] = 1.21, 95% confidence interval [CI]: 1.08 to 1.35). Another large population-based study obtained similar results, and it estimated the sleep score to assess sleep quality according to a previous study and indicated that a reduced sleep score led to increased KSD risk (hazard ratio [HR] = 1.07, 95% CI: 1.05 to 1.10) (45, 46). A prospective study in China measured sleep quality using the Pittsburgh Sleep Quality Index (PSQI) and KSD prevalence, and the PSQI was positively correlated with KSD prevalence (OR=1.18, 95% CI: 1.08 to 1.28) (47). Another sleep disturbance, obstructive sleep apnea (OSA), is common, with an incidence of 4.0% to 32.8% for middle age and 22.4% for older than 60 years (42), and has been confirmed to be a risk factor for KSD (HR=1.35, 95% CI: 1.23 to 1.48) (48). A retrospective study including 127 patients performed a univariable comparison of 24-h urine components and found that OSA is related to changes in urinary analytes that promote KSD (49).

**Figure 2:**
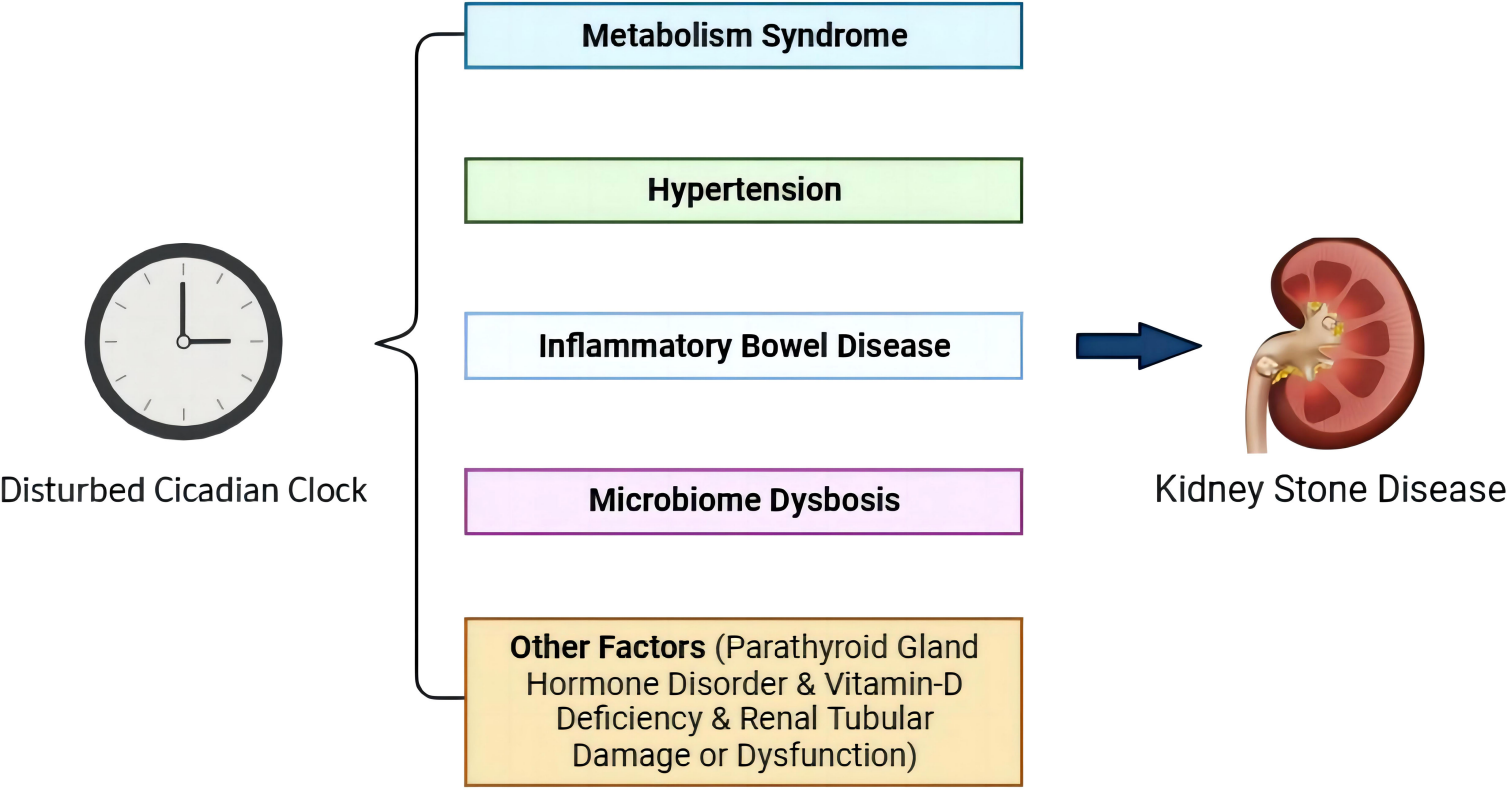
Involvement of circadian rhythm disturbance in KSD.

Although several studies have elucidated that disturbed circadian rhythm can induce KSD, the endogenous mechanism is poorly understood. KSD is a multifactorial systemic disease with diverse triggers. Based on epidemiological studies, metabolic syndrome, hypertension, inflammatory bowel disease, microbiome dysbiosis, parathyroid hormone disorder, and vitamin D deficiency can induce KSD, and they are related to a disrupted circadian clock (50–52) (**Figure 2**).

## 5 Association between circadian rhythm disturbance and KSD

Sleep and circadian rhythm disturbance is associated with alterations in circadian gene expression, and mammals with specific circadian gene deficiencies or mutations exhibit abnormal sleep/awake rhythms (1, 53–55). Here, we comprehensively summarize some potential links between circadian rhythm disturbance and KSD. The factors related to KSD that are affected by sleep and circadian rhythm disturbance and the mechanisms are described as follows.

### 5.1 Glycolipid metabolism disorder (metabolic syndrome)

Metabolic syndrome (MetS) is a cluster of conditions that usually occur together to increase the risk of T2D and CVD, including hypertension, chronic hyperglycemia (T2D and impaired glucose tolerance [IGT]), and dyslipidemia (obesity, decreased high-density lipoprotein [HDL] level, increased low-density lipoprotein [LDL] or total cholesterol [TC] level), and its prevalence is approximately 25% of adults with an increasing trend at advanced ages (56, 57). MetS is confirmed to be an independent risk factor for KSD (OR=1.49, 95% CI: 1.26 to 1.76) (58, 59). The pathological mechanism of MetS in KSD mainly consists of insulin resistance (IR), hyperglycemia, and vascular dysfunction (59, 60).

MetS is significantly associated with circadian rhythm disturbance. A new concept, circadian syndrome (CirS), was suggested to indicate MetS related to disrupted circadian rhythm (61, 62). Xiao et al. first reported the relationship between CirS and KSD prevalence in a cross-sectional study and obtained similar results (OR=1.42, 95% CI: 1.06 to 1.91) (23). A longitudinal analysis from Canada investigated 393 participants for a mean 6-year follow-up and found that short sleepers were at significantly higher risk of MetS (relative risk [RR]=1.74; 95% CI: 1.05-2.72) (63). The large population-based study by Bayon et al. revealed that shift work was correlated with higher MetS risk (OR=4.45, 95% CI: 1.36 to 14.56) (64). The cross-sectional data from the New Hoorn Study cohort indicated that SJL was correlated with MetS with a prevalence ratio of 1.62 (65). Additionally, OSA can cause MetS and increased waist circumference (WC) and triglyceride (TG) levels in MetS can induce OSA (66, 67). Here, we reviewed the effects of MetS on KSD from two aspects.

#### 5.1.1 Type 2 Diabetes (T2D)

The KSD prevalence was higher in T2D patients than in healthy controls (21% *vs*. 8%, p < 0.05), and the recurrence rate was twice as high (2.1 *vs*. 1.3, p < 0.05) (68, 69). A cohort study showed that the population with T2D had a higher KSD risk (OR=2.44, 95% CI: 1.84 to 3.25) (70). IR can hamper the ability of ammonia genesis to respond to acid load in the kidney, which results in hyperacid urine and elevated urinary calcium excretion by compensatory hyperinsulinemia (60, 68). Oxidative stress (OS) is another link between T2D and KSD; increased reactive oxygen species (ROS) generated by disordered glycometabolism, oxidative damage of β-cells in the pancreas, and endothelial dysfunction cause OS and inflammation in RTECs, resulting in increased levels of superoxide dismutase and malondialdehyde and decreased levels of antioxidants (71). These factors eventually lead to KSD.

Glycometabolism is a complicated physiological process and presents a significant diurnal oscillation. Variations in both the secretion and sensitivity of insulin display obvious circadian rhythms, and glucose tolerance (GT) is higher in the morning than in the evening in healthy people, but this oscillation disappears in T2D patients and IGT appears when the circadian oscillation is damaged (72–74). Many studies have revealed that the risk of IR and T2D is higher in populations with sleep disorders, SJL, and shift work, and it may be reduced by extended sleep duration and circadian readjustment strategies (11, 75). Cui et al. investigated the association between sleep duration and the risk of T2D in a case-control study and found a U-shaped association (≤ 6 h, OR = 1.74, 95% CI: 1.01 to 3.01; 8-9 h, OR = 1.46, 95% CI: 1.04 to 2.06) compared to sleep duration of 6-8 h, in which abnormal sleep duration increased the T2D risk (76). A prospective study including night workers and day workers with 4 years of follow-up showed that the risk of obesity and T2D was 5 times higher in night shift workers than in daytime workers (77). Then, a meta-analysis assessed the association between shift work and T2D and revealed that shift work increased the risk of T2D (RR=1.10, 95% CI: 1.05 to 1.14), and the trend was similar in the population with SJL (78, 79).

The possible relationship between disrupted circadian rhythm and T2D has been clarified by numerous studies. Rodents with SCN dysfunction display global or partial circadian gene disruption and easily developed IGT, hyperglycemia, hyperinsulinemia, decreased insulin secretion and sensitivity, and β-cell defects in the pancreas (80–82). *Clock* mutant mice show hyperglycemia, decreased levels of expression, and phase shifts of RNA oscillation of genes that participated in glucose sensing, insulin signaling, islet growth, and development (83, 84). It also disrupts hepatic glycogen oscillation and changes the circadian mRNA and protein expression of glycogen synthase 2 (limiting enzyme in glycogenesis) in mice (85). Whole-body *Bmal1* knockout (KO) obliterates the systemic insulin sensitivity rhythm to develop increased fasting blood glucose, glucose intolerance, and hypoinsulinemia (83, 86). Severe IGT, increased glycemia levels, and decreased insulin secretion are found in pancreas- or β-cell-specific *Bmal1* KO mice (87). The whole-body *Cry1/2* KO severely damages glucose homeostasis with IGT (88). Another study by Zhang et al. demonstrated that the hepatic overexpression of *Cry1* results in decreased gluconeogenesis and lower glycemia in diabetic mice (89). Qian et al. investigated *Per1*:LUC transgenic rats exposed to light during the night for 10 weeks and showed disruption of islet circadian clock through impairment in the amplitude, phase, islet synchrony of clock transcriptional oscillations, and diminished glucose-stimulated insulin secretion (90). Furthermore, *Per2* inhibition decreases glycemia levels and gluconeogenesis and stimulates insulin secretion (91). Then, global *Rer-erbα*/*β* dual KO mice exhibit hyperglycemia by disturbing the insulin signaling pathway (92). Meanwhile, *Rev-erbα* mutant mice fed a chow diet have slight hyperglycemia without IR, which can be explained by *REV-ERBα* affecting glycemia by regulating glucose-6-phosphatase and phosphoenolpyruvate carboxylase (93).

In summary, primary findings indicated that a disturbed circadian rhythm leads to T2D mainly by regulating enzymes and insulin in glycometabolism. However, direct evidence about how circadian disorders affect T2D and KSD remains unclear.

### 5.2 Lipid abnormalities and obesity

Lipid metabolism disorders are common in KSD patients. A retrospective study enrolled 2242 patients with KSD and found that high TC levels were significantly associated with higher UAS risk (p=0.006), and the 24-h urine analysis presented a significant positive correlation between low HDL levels and lower urine pH and higher urinary oxalate and uric acid levels (94, 95). A demonstrably increased KSD risk in the obese population was observed in a cohort study, and in particular, an increased level of visceral adiposity was a risk factor for hypercalciuria and UAS (OR=3.64, 95% CI: 1.22 to 10.85) (96, 97). Obesity promotes systemic inflammation and OS, leading to tissue immune cell infiltration and contributing to stone formation. It facilitates the expression of adipokines and some inflammatory molecules, such as tumor necrosis factor-α and interleukin-6, which were detected in the renal tissue and urine of KSD patients (98). Furthermore, calcifications by lipid deposition within the hyperosmotic turbulent vasa recta erode into the nearby collecting system and interstitium to promote RP formation, which is further confirmed by the presence of cholesterol identified in stones and renal papillary (95).

Lipid metabolism is precisely regulated by the circadian clock. Plasma lipids present a day-night variation within a narrow range independent of food intake, with the peak level of HDL in the early rest phase and a decrease in the active phase (99, 100). Circadian rhythm disturbance causes lipid abnormalities and obesity. A study investigated the association between sleep duration and obesity in children and adolescents and revealed that short sleep duration increases the risk of obesity (OR=1.69, 95% CI: 1.25 to 2.29) and elevated WC (OR=1.49, 95% CI: 1.13 to 1.97), which is similar to the study by Brocato et al. (101, 102). Another longitudinal investigation including 815 workers showed that workers with greater SJL are more likely to be obese (OR=1.20, 95% CI: 1.00 to 1.50) (103). In addition, circadian rhythm disturbance alters the plasma lipid profile by increasing the levels of cholesterol, TGs, and LDL and decreasing the level of HDL (104, 105).

Clock gene expression alteration has been comprehensively verified in both human and animal models. Vieira et al. analyzed the 24-h pattern of clock gene expression in an obese population and showed that the expression of *CRY2* and *REV-ERBα* was upregulated in obese participants. A positive correlation was observed between *REV-ERBα* expression and body mass index and WC in the obese population. The expression of *CLOCK* was positively correlated with LDL and *RORα* with HDL levels. Obese people with MetS presented a positive correlation between *PER2* expression and LDL, while *REV-ERBα* was correlated with WC. *CRY2* and *REV-ERBα* are considered clock genes upregulated in obesity (106). Hepatic *PER2*, *PER3*, and *CRY2* showed lower expression in the obese groups than in the normal control group (107). In animal experiments, the diurnal feeding rhythm is significantly impaired in homozygous *Clock* mutant mice, and they are more likely to develop hyperphagia, obesity, hyperleptinemia, and hepatic steatosis (80, 83). Microsomal TG transfer protein levels in the enterocytes of *Clock* ^Δ19/Δ19^ *Apoe* ^−/−^ mice are higher, and enterocytes secret more chylomicrons. Clock^Δ19/Δ19^ protein enhances intestinal cholesterol absorption, as well as the secretion of chylomicrons and cholesterol (108). The *Bmal1* KO mice display ectopic body and liver fat formation, hyperlipidemia, increased circulating leptin levels, and the absence of glucose fluctuation, also presenting earlier signs of obesity under a high-fat diet (HFD) (86, 109). In liver-specific and global *Bmal1^−/−^* mice, an elevation of circulating free fatty acids and higher TG formation is detected and can be reversed by *Bmal1* overexpression (110). Adipose-specific *Bmal1^−/−^* mice show increased weight gain and fat formation with increased calorie intake during the daytime (84). *Per1/2/3* triple-deficient mice are more likely to be obese, suggesting a potential role in body weight regulation (111). Moreover, *PER1* is identified to bind with major hepatic enzymes in bile acid synthesis, and *Per1* expression can be enhanced to increase fat absorption and accumulation in mice (112). *Per2* KO mice gain an altered lipid profile and downregulated triacylglycerol levels, while *Per2* deficiency in fibroblasts can promote adipocyte differentiation via the direct interaction with PPARα/γ and their target genes (113, 114). *Per3* KO promotes adipogenesis *in vivo* by a clock output pathway in which *PER3* and *BMAL1* directly affect transcription factor Klf15 expression in adipocyte precursor cells (115). In addition, *Cry1/2* double null mice have abnormal serum and hepatic TG levels (88). Furthermore, *Rev-erbα* deficiency increases plasma lipid levels and decreases hepatic cholesterol and TG levels (116).

Although circadian genes in lipid abnormalities have been extensively studied, the pathological process in KSD deserves further research.

### 5.3 Hypertension

The kidney is the central organ that regulates blood pressure (BP). Poorly controlled BP results in kidney diseases and influences BP regulation in positive feedback (117). According to historical clinical investigations, hypertension (HTN) may correlate with KSD. Cappuccio et al. recruited 688 male workers in a cross-sectional study and found the relative risk of hypertensive participants having a history of KSD was twice that of the normal group (OR=2.11, 95% CI: 1.17 to 3.81), which is similar to the larger cohort study based on U.S. population by Hill et al. (RR=1.79, 95% CI: 1.19 to 2.71) (118). Data from a prospective cohort proved this finding (119, 120). A study confirmed that HTN can be an independent predictive determinant for recurrent KSD, especially in non-obese SFs (121). Inconsistently, the studies published by Madore et al. showed that the KSD incidence was comparable in both the hypertensive population and normal population (OR= 0.99, 95% CI: 0.82 to 1.21), and similar results were obtained when limited to middle-aged women (122, 123). The inconsistency of diagnostic criteria for HTN can lead to opposite results, and it is essential to remeasure the association in new criteria. Considering the widespread popularity of HTN and the link between HTN and metabolism, it cannot be ignored. Currently, the etiology of HTN in KSD consists of an alteration of urine components, IR, inflammation, and OS (124). Increased urine calcium excretion is caused by central volume expansion (the ‘central blood volume’ theory), and higher excretion levels of oxalate and uric acid were detected in hypertensive patients (119, 124, 125). Furthermore, ROS overgeneration by the activated renin-angiotensin-aldosterone system (RAAS) promotes RTEC injury and crystal formation (124, 126).

BP in healthy individuals exhibits precise daily variation, which is characterized by an increase after awakening followed by a decrease at night during sleep (1, 127). Disturbed circadian rhythm eliminates the diurnal rhythm of BP, resulting in elevated BP that transfers to HTN (127–129). Grandner et al. analyzed more than 700,000 adults from two large cohorts to illustrate that the HTN risk was higher in sleep deficiency compared to 7 h (≤ 4 h: OR=1.86, ≤ 5 h: OR=1.56, ≤ 6 h: OR=1.27, p < 0.0005 for all) (130). A dose-response meta-analysis found a higher HTN risk for shorter sleep duration, and previous meta-analyses reported similar results to strengthen this association (47, 131). For OSA patients, the HTN risk was higher than that in healthy controls (OR=2.84, 95% CI: 1.70 to 3.98) and was positively correlated with the OSA grade (132). Additionally, night shift workers have a higher HTN risk than normal controls, which increases with an increasing frequency of night shift work (133). Although there is no significant association between SJL and HTN in historical studies, it is worth noting that a recent study presented a morning BP surge caused by acute SJL (134, 135).

The underlying mechanism of disturbed circadian rhythm in HTN is complicated. First, in OSA patients, sympathetic nervous system overactivity, disruption by OS, and inflammation in vascular structure and functions contribute to the abnormal diurnal pattern of BP (132, 136). Intermittent hypoxia (IH) and negative pressure against obstruction activate adrenal, renal, and peripheral chemoreceptors to increase the circulating levels of hormones such as renin and catecholamine and decrease nitric oxide (NO) synthesis, leading to upregulated sympathetic system activity (136, 137). The interactions between the sympathetic system and the kidney secondarily activate RAAS to increase BP (138). Additionally, IH disrupts endothelial NO expression by promoting ROS generation. Endothelial dysfunction is mainly caused by inhibited NO bioactivity and bioavailability impairs vascular vasodilation and enhances vasoconstriction (25, 136).

Circadian gene abnormalities also trigger HTN. *Clock* mutation represses the expression level of *Atp1b1*, which encodes the β1 subunit of the Na^+^/K^+^-ATPase to elevate BP (139). In hypertensive rodents, myeloid-specific deficiency of *Bmal1* exacerbates vascular remodeling and accelerates HTN formation by influencing the profibrotic macrophage phenotype (140). A human study showed a higher level of *Per1* mRNA in the renal medulla in the hypertensive group than in the normal control group, suggesting a role for *Per1* in the regulation of BP by renin (141). Doi et al. examined BP regulation in global *Cry1/2* double null mice and revealed that *Cry1/2* KO mice developed salt-sensitive HTN compared to wild-type (WT) mice (142). Surprisingly, KO or mutation of circadian genes also causes the absence of diurnal rhythm in BP and significantly decreased BP (127, 128, 143). To explain this, glycolipid metabolism disorders should be considered due to the close link between HTN and MetS (143, 144). Moreover, the relationship between circadian genes and the proteins expression in the local kidney that play roles in water and electrolyte balance is inspiring. The sodium chloride (NaCl) co-transporter (NCC) is involved in sodium reabsorption and BP maintenance and Richards et al. proved that *Per1* inhibition reduces NCC expression and results in lower BP in mice (145). Zuber et al. also investigated the intrinsic circadian rhythm system and found that *Clock* mutant mice exhibit significant alterations in the renal expression of several key regulators of water or sodium balance (vasopressin V2 receptor, aquaporin-2, aquaporin-4, epithelial sodium channel), which functionally leads to dysregulation of sodium excretion rhythms and a significant decrease in BP (146). In addition to circadian genes, serum- and glucocorticoid-induced kinase 1 (*SGK1*) in renal tubules, a clock-controlled and glucocorticoid receptor- and mineralocorticoid receptor-induced gene was recently shown to participate in BP circadian regulation. Staub et al. generated a tubular-specific *Sgk1* KO model and found that *Sgk1* deletion elevates pulse pressure by increasing the circulating aldosterone level and disrupts the BP rhythm (147).

Together, these studies indicated that circadian rhythm disturbance can disrupt BP homeostasis bidirectionally. However, since epidemiological studies focused on KSD and HTN are full of controversies, this hypothesis needs further verification.

### 5.4 Inflammatory bowel disease

Inflammatory bowel disease (IBD) is an autoimmune disease characterized by chronic intestinal granulomatous inflammation and includes two main types: Crohn’s disease (CD) and ulcerative colitis (UC) (148, 149). KSD is one of the most frequent extraintestinal manifestations of IBD, with a higher prevalence in IBD patients than in normal controls. In the cohort from Mississippi, 6% and 6.7% of CD and UC patients developed KSD, respectively, which is similar to the data in Switzerland (150–152). An observational study revealed that UC was a risk factor for KSD (OR = 4.2, 95% CI: 1.1-15), and the KSD prevalence in UC and CD was comparable (153). The pathological process of IBD in KSD was exhaustively reviewed by Corica et al. (152). Briefly, UA supersaturation by low urine volume and pH, hypercalciuria by bile salt malabsorption, increased colonic epithelium permeability to oxalate, and decolonization of *Oxalobacter formigenes* (*O. formigenes*) are radical.

IBD is strongly affected by circadian rhythm disturbance. The IBD incidence was significantly higher in patients with shorter sleep durations than in normal controls (HR = 1.51, 95% CI: 1.10 to 2.09) in a 10-year follow-up analysis (154). A retrospective study enrolled 115 IBD subjects and 76 healthy controls to measure chronotype, SJL and sleep debt (SD), which showed that later chronotype was negatively correlated with severe IBD (r = -0.209, p < 0.05) and that SJL was higher in the IBD group than in the controls (1.32 h ± 1.03 *vs*. 1.05 h ± 0.97, p < 0.05), while SD was also elevated in the IBD group compared to the controls (21.90 min ± 25.37 *vs*. 11. 49 min ± 13.58, p < 0.05) (155).

In IBD patients and animals, abnormal expression and status of clock genes are considered initial manifestations. Weintraub et al. analyzed clock genes in peripheral blood and intestinal mucosa and found that the expression levels of clock genes (*CLOCK*, *BMAL1*, *CRY1*, *CRY2*, *PER1*, and *PER2*) were significantly lower in both inflamed intestinal mucosa and leukocytes than in healthy controls, which was also reported in different tissues (peripheral blood monocytes, colon) (156–158). Rodents with artificially induced colitis displayed decreased *Per2*, *Cry1*, *Rev-erbα*, and *Npas2* levels and increased *Rorα* in colon tissue (159). Kyoko et al. measured the tight junction proteins occludin and claudin-1 in *Clock* mutation ((*Clock* ^△19/△19^) mice to show that mice lacking *Clock* have persistently low levels of these two proteins and were more susceptible to intestinal injury (160). Similarly, *Bmal1* KO mice show worse UC and the absence of time-dependent variation in disease activity compared to *Bmal1^+/+^* controls, and epithelial proliferation in the colon presents a daily rhythm in *Bmal1^+/+^* controls but is absent in the *Bma1 KO* group, resulting in poor regeneration (161). Mice lacking *Rorα* or *Bmal1*-driven Lnc-UC (a long noncoding RNA that is associated with colitis, particularly by reducing *Rev-erbα* expression) are more likely to have colitis than the control group. Lnc-UC deactivates the activity of NLR family pyrin domain (NLRP) 3, which is essential in the induction of proinflammatory cytokines (159, 162, 163). *Bmal1* KO also leads to a lower level of regulatory B cells in the intraepithelial region, which expresses highly programmed death ligand 1 to alleviate colitis severity (164). Then, colitis is more severe in *Per1/2* KO mice than in WT mice, with decreased Paneth cells, goblet cells, lysozyme transcripts, and lysozyme proteins (165). Oh et al. presented that intestinal epithelial-specific *Rorα* KO leads to severe inflammation by reducing the level of Ki67, a cell proliferation marker, and p-DRP1, a molecule active in ATP production (162). All the evidence indicates that IBD is related to abnormal expression of circadian genes.

All these studies demonstrated that circadian rhythm disturbance can promote KSD via IBD. However, further research is necessary to better clarify the internal molecular pathways in the process of KSD.

### 5.5 Microbiome dysbiosis

Microbiomes coexisting with humans are important in maintaining health and causing diseases. The gut microbiome (GMB) is a large set of microorganisms that colonize our digestive tract, and the diverse mixture of bacteria within the genitourinary tract (often at low levels) is defined as the urinary microbiome (UMB) (166–168). Dysbiosis of GMB and UMB contributes greatly to KSD (168). The colonization rate of *O. formigenes* in CaOx stones has attracted great attention. Several studies have proven that the colonization rate of *O. formigenes* is lower in SFs than in controls (169–171). Stern et al. studied the distinct GMB in SFs and non-SFs and revealed that *Bacteroides* was 3.4 times more abundant in SFs than in non-SFs (34.9% *vs*. 10.2%, p = 0.001), and *Prevotella* was 2.8 times more abundant in non-SFs than in SFs (34.7% *vs*. 12.3%, p = 0.005). In urinary analysis, *Eubacterium* was negatively correlated with oxalate levels, and *Escherichia* tended to have an inverse correlation with citrate levels (172). Compared to GMB, UMB in KSD is underexplored due to dramatic variation (168, 173). A case-control investigation profiling the UMB in male patients with calcium-based stones found that the UMB diversity was markedly lower than that in healthy controls, and the components were also different in the two groups (p < 0.001). The taxa at the genus level that significantly differentiated the two groups were *Prevotella* in the normal group and *Acinetobacter* in the KSD group (174). A meta-analysis including 8 studies indicated that the abundance of *Bacteroides*, *Lactobacillus*, and *Prevotella* showed the most significant difference in GMB between KSD patients and healthy controls (175). In UMB, *Escherichia coli* (*E. coli*), *Lactobacillus*, *Staphylococcus*, *Streptococcus*, and *Klebsiella* are considered vital in KSD based on evidence *in vivo* and *in vitro* (166, 168, 176–178). The pathogenic mechanism of GMB is poorly understood, oxalate accumulation by oxalate degradation dysfunction and related metabolic disorders is widely recognized (166, 179). Meanwhile, urease enzymes and inflammation are also important (16, 180).

Microbiomes have been found to follow a strict circadian rhythm (167, 181). Up to 60% of the total microbial composition oscillates rhythmically, which translates to diurnal fluctuations in 20% of commensal species of the GMB in mice and 10% in humans. The GMB abundance of ad libitum-fed mice under a strict light-dark cycle was measured at the changing point, and significant diurnal fluctuations were identified in the abundance of more than 15% of the GMB (182). Circadian misalignment disrupts GMB homeostasis and causes diseases. Smith et al. explored the correlation between GBM diversity and sleep physiology to show that diversity was positively correlated with sleep efficiency and duration. Sleep duration reduction can significantly decrease GBM diversity (183, 184). Additionally, exposure to SJL exacerbated GBM and metabolite homeostasis in the jejunum and colon of mice and was also detected in humans (181, 185).

Studies support that clock gene expression alterations contribute to GMB dysbiosis. Compared to WT mice, *Clock* mutant (*Clock* ^Δ19/Δ19^) mice have a decreased *Firmicutes*/*Bacteroidetes* ratio, especially when paired with alcohol consumption or HFD (186). Liang et al. constructed *Bmal1* KO mice and found that *Bmal1* KO abolishes the oscillation and composition of GMB with a decreased abundance of *Prevotella* and an increased abundance of *Bacteroides* (187). In *Per1/2*-deficient mice, *Bacteroides* and *Lactobacillus* lost oscillations in relative abundance (182). Furthermore, in the interaction between GBM and MetS, dysbiosis significantly promotes the development of MetS. A high-sugar diet and HFD can aggravate the impact of circadian disorganization on GBM and further disturb glycolipid metabolism (188–190). In UMB, studies are limited. The diurnal oscillation in *Streptococcus pneumoniae (S. pneumoniae*) is driven by external clues, such as temperature (191). *Per1* mutant flies are more sensitive to *S. pneumoniae*, and the elevated infection sensitivity can be a consequence of the circadian regulation disturbance of phagocytosis in these fly mutants (192, 193). In *E. coli*, circadian rhythms are driven by special genes, including *radA* and *KaiC* (194). Furthermore, a red and blue photoreceptor was contained in *E. coli* to adapt to the day-night cycle, which may provide evidence for dysbiosis in abnormal light exposure by circadian disturbance (195).

These studies provide a preliminary indication of the correlation between MB and KSD. However, data from mammals are still necessary, and since there is an interaction between GMB and UMB, further research is needed.

### 5.6 Other factors

#### 5.6.1 Parathyroid gland hormone disorder

Disordered calcium metabolism contributes greatly to KSD. Goodman et al. concluded that the risk of hypercalcemia was 9 times higher in KSD patients than in normal controls (196). Parathyroid hormone (PTH) is essential to enhance calcium reabsorption and inhibit phosphorus reabsorption in the kidney (197). Elevated PTH with hypercalcemia in primary hyperparathyroidism (PTHP) is a well-recognized reason for KSD (198). An observational study revealed that parathyroidectomy was effective in the therapy of KSD recurrence by idiopathic hypercalciuria and that stone activity significantly decreased after surgery (0.05-0.15 *vs* 0.50-0.75, p < 0.001) (199, 200).

The PTH level exhibits a bimodal pattern over 24 hours, with a maximum peak in the afternoon and a small peak at night (201). Circadian impairment leads to abnormal PTH levels. Higher serum PTH level was observed in patients with moderate OSA and severe OSA than in healthy controls (63.11 ± 36.11 and 53.16 ± 25.29 *vs* 43.71 ± 24.45, p < 0.05) (202). Then, Sleep disturbance is common in PTHP patients, but the causation is unclear (203, 204).

Clock genes in the parathyroid gland (PTG) were explored, and their disturbance caused parathyroid gland dysfunction (205). Normal circadian clock operation was confirmed in animal models with a periodicity of 24 hours and was significant for *Bmal1*, *Npas2*, *Per1,2,3*, *Cry1,2*, and *Rev-Erbα*. In hyperplastic PTG tissue, circadian genes were deregulated, with significant upregulation of *Per1,2* and *Rev-Erbα* and downregulation of *Npas2* (206). Egstrand et al. investigated the alteration of circadian genes during a 24-h cycle in murine PTG and found rhythmic expression of parathyroid signature genes, and this rhythm is essential for PTG function regulation. Mice with PTG-specific *Bmal1* knockdown (PTHcre;*Bmal1* ^flox/flox^) were created, and a global decrease in circadian genes was observed, including *Clock*, *Npas2*, *Cry1,2*, and *Per1,2,3*. Compared to WT, PTHcre;*Bmal1* ^flox/flox^ shows a higher parathyroid cell proliferation response and led to PTHP (205). The identification of transcriptional patterns in human PTG tissues presents that the transcript expression levels of *PER1* and *CRY1/2* are significantly lower in PHPT tissue than in healthy tissue (207).

Taken together, these studies indicate that circadian gene disturbance leads to PTG disorder and is a risk factor for KSD. However, more investigations are needed.

#### 5.6.2 Vitamin D Deficiency

Vitamin D (vit D) participates in maintaining calcium homeostasis, and vit D deficiency (VDD) is defined as a serum 25-hydroxyvitamin-D [25(OH)D] concentration less than 30 ng/ml (208). VDD affects a large population globally and is more prevalent in KSD patients (209). The investigation by Elkoushy et al. showed that more than 80% of KSD patients have VDD, which is consistent with a multicenter study (210, 211). A case-control study in Spain including 366 participants found that calcium SFs had lower levels of Vit D (25.7 *vs*. 28.4 ng/ml, p = 0.02) and a higher percentage of VDD than non-SFs (28.0% *vs*. 15.7%, p = 0.009) (212). Vit D is a fundamental regulator of systemic inflammation, OS, and mitochondrial respiratory function. Based on current studies, cell injury by OS and inflammation by overproduced ROS are the cardinal pathogenic factors for KSD in VDD (213, 214).

Vit D status is significantly influenced by a circadian rhythm in which serum Vit D presents a significant diurnal rhythm with a nadir in the morning and is followed by a rapid increase to a plateau during the day (215). VDD is widespread in populations with abnormal light exposure or insufficient sleep (216). Piovezan et al. indicated that short sleep duration showed an independent association with VDD (OR = 1.61, 95% CI: 1.25 to 2.26) (217). Then, shift workers are more likely to develop VDD. An investigation in Italy measured serum 25(OH)D in workers and observed that the level was lower in night shift workers than in daily workers (13.4 ± 5.3 ng/mL *vs*. 21.9 ± 10.7 ng/mL, p < 0.001) (218, 219).

Endogenous vit D is synthesized in the skin from the cholesterol-like precursor (7-dehydrocholesterol) present in epidermal cells by exposure to ultraviolet B (UV-B) from sunlight (220). Therefore, a lack of UV exposure reduces vit D levels and exacerbates OS in RTECs, which leads to KSD (129, 218). Moreover, research on the interaction between VDD and circadian genes is limited. Kwai et al. created an intestinal *Bmal1* KO model (*Bmal1*_Int_-/- mice) and found that the vit D receptor (VDR) target genes in the intestine are disrupted. The expression of VDR and *Vdr* peaks at ZT8 (*zeitgeber* time [ZT]: light on, ZT0–ZT12; lights off, ZT12–ZT24) in the control group but disappears in *Bmal1*_Int_-/- mice. The experiment in Caco-2 cell lines also reveals that the *BMAL1* KO reduced *VDR* and VDR expression (221). However, other circadian genes are still unclear in VDD, which is worth more analysis.

#### 5.6.3 Renal Tubular Damage or Dysfunctions

The combination of urinary supersaturation and renal tubular damage is vital in stone formation. Renal tubular damage is related to sleep disorders, especially OSA (222, 223). The high oxygen demand of renal tubules makes them vulnerable to hypoxia by chronic IH in OSA and easily advanced to renal tubular injury (223). However, such a theory needs to be backed up by more research, and its relationship with KSD should be further elucidated based on direct evidence.

Distal renal tubular acidosis (dRTA) syndrome is a condition caused by the acidification defect in the collection tubule and the inability of the distal nephron to maximally increase the urinary secretion of protons ([H+]) in the presence of metabolic acidosis, characterized by a persistent hyperchloremia, normal plasma anion gap and metabolic acidosis with a relatively normal glomerular filtration rate. Patients with dRTA have elevated urinary calcium, recurrent CaOx or CaP stone formation, and nephrocalcinosis (224, 225). In addition to inherited dRTA, secondary dRTA is caused by numerous triggers, including autoimmune diseases, nephrotoxins, and miscellaneous aetiologies. Currently, there are insufficient epidemiological and basic studies to confirm the relationship between dRTA and circadian rhythm disturbance.

## 6 Future perspective

Growing evidence indicates that some therapeutic strategies enhancing circadian clock function or circadian gene expression may be beneficial for the prevention of KSD. First, feeding time is one of the most important external *Zeitgebers* in peripheral tissues, and unhealthy feeding time promotes diseases. A systematic review suggested that fasting results in altered urine metabolites and density, although this did not transfer to clinical outcomes. Safe fasting practices are vital for high-risk patients to prevent KSD (226). For OSA patients, continuous positive air treatment significantly reduces tubular damage, which may decrease the KSD risk (223). Moreover, melatonin (Mel), a hormone released from the pineal gland against SJL and sleep disorders with anti-inflammatory and antioxidative functions, can prevent crystalluria and kidney damage caused by crystal formation and aggregation (227, 228). Song et al. found that Mel has protective effects on oxalate-induced endoplasmic reticulum stress and apoptosis via the activation of the adenosine 5’-monophosphate-activated protein kinase pathway in HK-2 cells (229). In addition, *BMAL1* is a therapeutic target *in vitro*. *BMAL1* overexpression stimulated the OS-related NRF2/HO-1 pathway to reduce CaOx stone formation. This suggests that maintaining normal rhythms and properly intervening in some related circadian genes and downstream antioxidant pathways may benefit the prevention of CaOx stones (230). According to current limited studies, it can be speculated that artificial interventions in sleep status and circadian rhythm have the potential to prevent KSD. However, more clinical and basic research is needed.

## 7 Conclusions

Since KSD is a major challenge for global health, its potential mechanism should be investigated. Increasing convincing evidence has elucidated that a disordered circadian clock is a putative factor for KSD. This review summarizes the biological characteristics of the circadian rhythm, the mechanism of KSD, and the putative mechanism of the circadian rhythm disturbance in KSD. Existing clinical and basic studies have indicated that circadian rhythm-based interventions have potential clinical value in the management of KSD, but the specific and accurate mechanism of KSD caused by circadian rhythm disturbance is still unclear. KSD is not an isolated kidney disease, but a systemic disorder affected by various factors. Understanding the relationship between circadian rhythm and systemic multi-organ and multi-system health status is essential. In addition, both behavioral and pharmacological interventions related to rhythm modification deserve more research. A comprehensive and in-depth exploration of the mechanism of KSD caused by sleep and circadian rhythm disturbance and the efficacy of chronothrapies for KSD are necessary and can provide a new strategy for the clinical management of KSD.

## Author contributions

SH: Conceptualization, Writing – original draft. JHW: Writing – original draft. TL: Writing – original draft. SY: Data curation, Writing – review & editing. JC: Writing – review & editing. YT: Supervision, Writing – review & editing. YX: Data curation, Writing – review & editing. JW: Conceptualization, Writing – review & editing. YB: Conceptualization, Writing – review & editing.
